# Mediating roles of attention-deficit/hyperactivity disorder symptom severity and self-control on the relationship between adverse childhood experiences and adult aggression

**DOI:** 10.1007/s00702-025-03010-1

**Published:** 2025-09-23

**Authors:** Johannes Merscher, Wolfgang Retz, Petra Retz-Junginger, Steffen Barra

**Affiliations:** 1 Institute for Forensic Psychology and Psychiatry, Saarland University, Homburg, Germany; 2 Department of Psychiatry and Psychotherapy, University Medical Center of Johannes Gutenberg University Mainz, Mainz, Germany

**Keywords:** ADHD, Self-control, Adverse childhood experiences, Aggression

## Abstract

Previous research has identified attention-deficit/hyperactivity disorder (ADHD), adverse childhood experiences (ACEs), and low self-control as risk factors for aggressive behavior. While associations among these variables have been widely documented, the underlying mechanisms remain unclear. Grounded in the General Theory of Crime, this study investigates the relationship between ACEs and aggression, with a particular focus on the mediating roles of ADHD and self-control in a serial mediation model. Based on the theoretically grounded assumption that ACEs contribute to adult ADHD symptomatology, which impairs self-control and predicts aggressive behavior, this study examines the dynamic interplay among these constructs in a mixed-gender sample of 350 participants aged 18 to 76 years (M = 34.9, SD = 13.1). Serial mediation analyses revealed positive associations between ACEs and aggression, as well as between ACEs and ADHD. Additionally, a significant positive correlation was observed between ADHD and aggression. Conversely, self-control exhibited a negative relationship with both ADHD and aggression. Mediation analyses indicated that ADHD and self-control partially mediated the link between ACEs and aggression in adults irrespective of gender and age. Our results suggest that violence prevention efforts could benefit from early interventions aimed at reducing ACEs, diagnostic assessment and treatment of ADHD symptoms, and therapeutic interventions designed to enhance self-control and thereby mitigate their impact on aggression.

**Supplementary Information:**

The online version contains supfplementary material available at 10.1007/s00702-025-03010-1.

## Introduction

Attention-deficit/hyperactivity disorder (ADHD) is one of the most prevalent psychiatric disorders in childhood and adolescence but also highly relevant in adults (Chaulagain et al. 2023; Popit et al. 2024; Sayal et al. 2018). An estimated 2.58% of adults met criteria for persistent ADHD since childhood, whereas 6.76% exhibited current ADHD symptoms of clinical relevance in adulthood (Song et al. 2021). The disorder is characterized by a broad spectrum of functional impairments that impact professional and social functioning (Faraone et al. 2021), leading to sustained distress and adversely impacting quality of life (Cherkasova et al. 2022; Di Lorenzo et al. 2021; Ward et al. 2007). ADHD symptoms are associated with lower academic achievement (Arnold et al. 2020; Mannuzza et al. 1993), a pattern that may contribute to an increased risk of criminal involvement (Watts 2018).

The ADHD core symptoms of inattention, impulsivity, and hyperactivity, along with the severity of this behavioral disorder, have been linked to diverse types of aggression, violence, and delinquency, such as intimate partner violence and enhanced risk of on-going criminal behaviors (Arrondo et al. 2023; Barra et al. 2022; Merscher et al. 2025; Mohr-Jensen and Steinhausen 2016; Philipp-Wiegmann et al. 2018; Pratt et al. 2002; Retz et al. 2021; Saylor and Amann 2016; Wojciechowski 2021; Wymbs et al. 2012). Individuals with ADHD exhibit deficits in emotion regulation (Reimherr et al. 2005; Retz et al. 2012), which are also related to aggressive behavior (Anker et al. 2021). Accordingly, individuals with ADHD show disproportionately high rates of arrests, convictions, and incarcerations (Ångström et al. 2024; Young et al. 2015).

Assuming an impairment in anticipating the consequences of their actions (Bloomquist et al. 1997) and a diminished capacity to encode social cues in interpersonal interactions (Matthys et al. 1999), aggressive behavior in the context of ADHD has significant implications for an individual's social functioning and maladaptive social adaptation (Barkley 2002; Retz et al. 2021). Relationships with family members and peers are frequently affected (Gardner and Gerdes 2015). Meta-analytic findings by the research groups led by Faheem (2022) and by Faraone (2021) suggest that gender-specific differences exist in the prevalence and impact of ADHD in adulthood, with a higher prevalence observed in men than in women. In contrast, women appear to be more severely affected than men in terms of the impact of ADHD, particularly in areas such as social functioning and stress management. However, the gender gap appears to decrease from childhood to adulthood. In forensic samples, however, no differences between men and women were observed, even in adulthood (Young et al. 2015).

Adverse childhood experiences (ACEs) are severe early-life stressors in childhood and adolescence linked to long-term health risks, including chronic illness and early death, that can disrupt physical, cognitive, and neurological development (Larkin et al. 2014), and have also been linked to the development of ADHD symptoms (Alfonso et al. 2024; Crouch et al. 2021; Rodriguez et al. 2025). The prevalence of ACEs is significantly higher among individuals diagnosed with ADHD compared to healthy controls (Walker et al. 2021). Previous meta-analyses consistently demonstrated a robust association between ACEs and ADHD, assuming a dose–response relationship (Boswell et al. 2025; Craig et al. 2020; Szymanski et al. 2011; Wojtara et al. 2023; Zhang et al. 2022). This association may be explained by the fact that repeated stress exposure during early childhood can alter the course of neurodevelopment, setting an individual at risk of long-term cognitive and behavioral deficits (Hartman et al. 2019). Exposure to ACEs has been related to reduced gray matter volume in neural structures, including the prefrontal cortex, amygdala, and hippocampus (McLaughlin et al. 2019) and with increased neurite density index (NDI) in the corpus callosum (Hare et al. 2022). Chronic stress is associated with an increased risk of more severe and persistent ADHD symptoms, whereas a reduction in environmental stress has been linked to symptom attenuation (Hartman et al. 2019). Empirical research demonstrates that women are disproportionately affected by ACEs compared to men (Baglivio et al. 2014; Felitti et al. 1998). Beyond differences in overall prevalence, distinct sex-specific patterns have been identified with regard to the nature of ACEs. Women are more frequently exposed to sexual abuse, whereas physical abuse occurs more commonly among males (Baglivio et al. 2014; Dierkhising et al. 2019). While ACEs are associated with psychological distress in both sexes, their influence appears to be particularly salient for externalizing symptoms among females (Jones et al. 2022a, b).

Moreover, ACEs exhibit strong associations with aggressive behavior and violent crime (Braga et al. 2017; Burke et al. 2023; Fitton et al. 2020; Juan et al. 2020; Manzoni and Schwarzenegger 2019; Woehrle et al. 2022; Zhu et al. 2024). A recent meta-analysis (Stoppelbein et al. 2024) highlights a robust relationship between ACEs and both aggression and offending behavior across age groups (Malvaso et al. 2021). However, meta-analytic evidence (Wilson et al. 2009) indicates that effect sizes differ notably by study design, with cross-sectional studies yielding larger (d = 0.88) and longitudinal studies showing substantially smaller effects (d = 0.31). Assumptions explaining the ACE-aggression link include the individual's risk of imitating perceived aggressive behavior (Bandura 1973; Straus et al. 1980) but have also been based on neurological alterations that compromise social development and affect regulation (Solomon and Heide 2005; Teicher et al. 2003). Empirical findings indicate that, on average, males exhibit significantly higher levels of direct aggression, particularly physical aggression, than females. This gender difference has been consistently observed across various age groups and emerges as early as early childhood (Archer 2004). The greater propensity for indirect aggression observed in females, as reported by Eagly and Steffen (1986), appears to be limited to late childhood and adolescence (Archer 2004).

When investigating the links among ADHD, ACEs, and aggression, the role of low self-control must not be neglected. Due to its robust correlation with a broad range of criminal and deviant behaviors, low self-control has become one of the most rigorously examined constructs in behavioral and social sciences (Burt 2020; Pratt and Cullen 2000; Pusch 2024; Schoepfer et al. 2019). In their General Theory of Crime (1990), Gottfredson and Hirschi posit that the fundamental determinant of criminal behavior is a deficiency in self-control. Individuals exhibiting high levels of self-control are inclined to consider the long-term repercussions of their actions, whereas those with low self-control tend to disregard such consequences. Tangney et al. (2004) conceptualize self-control as the “ability to override or change one’s inner responses, as well as to interrupt undesired behavioral tendencies and refrain from acting on them”, aligning with Barkley’s (1997) definition of self-control as the capacity to regulate impulsivity and transient desires. Thus, self-control is commonly conceptualized as the inverse of reward-delay/choice impulsivity (Bullard et al. 2024). Both males and females possess largely comparable psychological structures and capacities for self-control. However, men may exhibit stronger antisocial or problematic impulses than women (de Ridder et al. 2012). A meta-analysis by Lv et al. (2025) supports this notion, demonstrating that males, depending on age, show a greater tendency to discount future rewards in favor of immediate gains.

Considering the associations between self-control, ACEs, aggression, and ADHD, previous research has indicated that the accumulation of ACEs may be a key factor in the lack of self-control (Meldrum et al. 2020; Perez et al. 2018). A recent gene × environment study found that ACEs impaired self-control, predicting elevated initial risk-taking followed by a sharper decline over time (Wang et al. 2025). Moreover, in the studies of Jones and colleagues (Jones et al. 2021; Jones et al. 2022a, b) an elevated number of ACEs was negatively associated with self-control in both male and female children and adolescents, with patterns characterized by high but delayed exposure, intermittent exposure, or chronically elevated exposure significantly exacerbating reductions in self-control. Furthermore, both cross-sectional and longitudinal studies exhibit robust correlations between self-control and physical violence in a meta-analysis conducted by Vazsonyi (2017). Eventually, meta-analytic findings suggest that children with ADHD exhibit moderate deficits in self-control compared to their typically developing peers whereas studies that include adult samples are scarce (Patros et al. 2016). Eme (2016) highlights that the responsibility of individuals with ADHD for their own behavior is mitigated by genetic and neurodevelopmental factors that impair self-control, restricting their behavioral and cognitive regulation. However, the influence of genetic and neurodevelopmental etiology has been overstated in responsibility assessments, while situational self-control strategies remain viable (Koi 2021).

Building on Thibodeau et al. (2015), recent studies have also examined how genetic mechanisms influence the associations among ACEs, ADHD symptoms, and later antisocial behavior (Meldrum et al. 2020; Perez et al. 2018). A polygenic index comprising DRD4, DRD2, DAT1, and COMT indicated that impulsivity partially mediated the maltreatment–antisocial behavior link, with moderation by genotype. These findings are consistent with earlier evidence showing increased ADHD symptoms and reduced self-control among maltreated children with the 10R/10R genotype (Li and Lee 2012; Wright et al. 2012). Again, comparable studies including adult samples are yet lacking.

In sum, prior research has almost consistently identified cross-sectional and longitudinal relationships among ADHD, ACEs, self-control, and aggression. However, to the best of our knowledge, no published study has examined all these dimensions simultaneously.

Building on a theoretically derived framework suggesting that ACEs contribute to adult ADHD symptomatology, which in turn impairs self-control and predicts aggressive behavior, this study examined the dynamic interrelations among these constructs. In light of the abovementioned research gaps, especially with regard to adult samples, the present study aimed at contributing to advanced research methodologies, forensic assessments, policy formulation, and practical interventions for individuals affected by ADHD.

It was hypothesized that the cumulative burden of ACEs would be positively related to adult aggression. Additionally, ACEs were assumed to be positively associated with ADHD symptom severity, which would be linked to reduced self-control, ultimately fostering adult aggression. Moreover, it was expected that ACEs would be directly associated with lower levels of self-control, while higher ADHD symptom severity would be associated with increased aggression. Exploratory gender-based analyses were conducted to determine whether the proposed serial mediation model would differ between women and men.

## Methods

### Procedures

This study was conducted as part of a broader investigation into clinically and forensically relevant risk factors for aggressive and other delinquent behavior within a heterogeneous sample of individuals assessed at the Institute for Forensic Psychology and Psychiatry, Saarland University, Homburg, Germany, which comprises out-patient clinic services for justice-involved individuals, as well as adults with suspected ADHD. As part of the standard assessment procedures, participants completed a set of self-report questionnaires, either in a paper-and-pencil format or via the online survey platform SoSci Survey (Leiner 2025), which included the measures relevant for this study. In addition, non-forensic/non-clinical participants were recruited by personal invitation. Informed consent was obtained, and participants could choose whether their anonymized data would be used for research purposes. Participation was voluntary and uncompensated. This study design aimed to enhance sample heterogeneity and maximize variance in the constructs of interest by including forensic, clinical, and non-forensic/non-clinical populations. The study was conducted in accordance with the ethical principles of the Declaration of Helsinki and received approval from the ethics committee of the Medical Chamber of Saarland, Germany (Protocol Code: 58/22).

### Participants

Between November 11, 2021, and February 26, 2025, a total of 429 individuals underwent assessment. Of these, 12 participants refused to have their data included in research analyses. Among the remaining 417 individuals, 350 completed the questionnaires relevant to the present study. The final sample consisted of 170 participants who identified as female (48.6%), 177 as male (50.6%), and 3 as diverse (0.9%). Due to the limited number of participants identifying as diverse, gender-specific analyses were conducted exclusively with male and female participants. However, data from diverse participants were retained for analyses in which gender was included as a controlled variable. Participants’ ages ranged from 18 to 76 years (M = 34.9, SD = 13.1). The sample comprised 105 individuals (30.0%) who had undergone forensic evaluation or therapy, 104 (29.7%) who had been assessed for ADHD, and 141 (40.3%) without forensic or clinical backgrounds.

### Measurements

#### ADHD

The severity of current ADHD symptoms was assessed using the German self-report questionnaire for adult ADHD (ADHS-SB; Rösler et al. 2021). The ADHS-SB consists of 22 items, each rated on a four-point Likert scale (0 = not applicable, 1 = slightly/occasionally, 2 = moderate/often, 3 = severe/almost always). The first nine items measure inattention, while the next nine assess hyperactivity/impulsivity. In sum, these 18 items form a total ADHD score. The remaining four items evaluate functional impairments and childhood symptomatology.

In addition to dimensional scores that describe symptom severity for inattention, hyperactivity/impulsivity, and total ADHD symptomatology, the authors suggest a cut-off value of 15 points on the total ADHD score to represent clinically concerning ADHD. However, for the current study, we focused on symptom severity as reflected by the dimensional total ADHD score.

The psychometric properties of the ADHS-SB have been previously validated by its developers. In the present sample, the questionnaire demonstrated excellent internal consistency, with Cronbach’s α = 0.963 for the total ADHD score.

#### Adverse childhood experiences

ACEs were assessed using the German 75-item version of the Maltreatment and Abuse Chronology of Exposure (MACE) scale (Isele et al. 2014; Teicher and Parigger 2015). Participants responded to a series of dichotomous yes–no questions, indicating whether they had encountered specific adverse events up to the age of 18 years. These events were categorized into ten distinct types: parental verbal abuse, parental non-verbal emotional abuse, parental physical abuse, emotional neglect, physical neglect, exposure to violence involving parents (either directed at or between them), exposure to violence against siblings, emotional abuse by peers, physical abuse by peers, and sexual abuse.

For this study, an ACE category was considered present if at least one item within it was endorsed, thus creating a total ACE score ranging from 0 to 10, reflecting the extent of polyvictimization. Prior research has demonstrated strong psychometric properties for both the original and the German adaptations of the MACE scale (Isele et al. 2014; Teicher and Parigger 2015). The reported types of ACEs were categorized according to the MACE subscales (see Fig. S1 in the Supplement).

#### Aggression

Aggressive behavior within the past 6 months was assessed using the aggressive behavior subscale of the German Adult Self-Report (ASR 18/59) by Achenbach and McConaughy (2003). The ASR 18/59 comprises 126 items evaluating various internalizing and externalizing problems, 15 of which constitute the aggressive behavior subscale. Items are rated on a 3-point Likert scale (0 = not true, 1 = somewhat or sometimes true, 2 = very true or often true). The psychometric properties of the ASR 18/59 have been validated in previous studies (Ivanova et al. 2015). In the present study, the aggressive behavior subscale showed high internal consistency (Cronbach’s α = 0.880).

#### Self-control

The assessment of self-control as a trait was conducted using a German translation of the Brief Self-Control Scale (BSCS; Sproesser et al. 2011). The scale comprises 13 items, with respondents indicating their level of agreement on a five-point Likert scale, ranging from 1 (not true) to 5 (exactly right). Higher scores may be indicative of a higher level of self-control. In the present study, internal consistency for self-control was high (Cronbach’s α = 0.884).

#### Covariates

In addition to the aforementioned variables of interest, age and gender identification were assessed as covariates.

### Statistical analyses

All statistical analyses were performed using IBM SPSS Statistics software for Windows, version 30.0.0.0. The general level of significance was set at *p* < 0.05 and all tests were executed two-sided. Internal consistency was classified as questionable with Cronbach’s α ≥ 0.60, acceptable with α ≥ 0.70, good with α ≥ 0.80, and excellent with α ≥ 0.90 (Blanz 2021). A multivariate analysis of variance (MANOVA) was employed for the purpose of descriptive group comparisons between male and female participants. The effect size partial eta^2^ (η_p_^2^) indicated a small effect with η_p_^2^ ≤ 0.01, a moderate effect with η_p_^2^ = 0.06, and a large effect with η_p_^2^ = 0.14 (Cohen 1973).

Furthermore, we examined (partial) correlations among the variables with coefficient cutoffs of |r|= 0.10 indicating small, |r|= 0.30 medium, and |r|= 0.50 large correlations (Cohen 2013). Correlation coefficients for male and female participants were compared using Fisher z-transformations (Weaver and Wuensch 2013). Both the MANOVA and correlation analyses were conducted using bias-corrected and accelerated bootstrapping with 5,000 resamples.

We further conducted serial mediation analyses utilizing Hayes' PROCESS macro (Hayes 2017; version 4.3.1) for IBM SPSS Statistics. We applied model 6 with z-standardized values to examine the effects of ADHD symptom severity and self-control on the relationship between ACEs and adult aggression. We included 5,000 bootstrap samples (with 95% confidence intervals) and robust standard errors (HC3: Davidson-MacKinnon) to ensure heteroscedasticity-consistent inference. Indirect effects were considered statistically significant if the confidence interval did not include zero.

## Results

### Descriptives

Table 1 presents the distribution of our variables of interest, overall and separated by gender. The MANOVA, which was controlled for age, revealed no statistically significant gender differences in the variables of interest, with the exception of a significantly higher severity of ADHD in women.

**Table 1:** Descriptive distribution of the total values of the variables of interest

	Total sample (N = 350)				Males (n = 177)				Females (n = 170)						
	M	SD	Range	Median	M	SD	Range	Median	M	SD	Range	Median	F(1,345)	*p*	η_p_^2^
MACE total score	4.30	2.69	0.00–10.00	4.00	4.15	2.48	0.00–10.00	5.00	4.48	2.89	0.00–10.00	4.00	1.28	0.263	0.00
ADHS-SB	17.05	13.71	0.00–53.00	13.00	15.63	13.02	0.00–50.00	13.00	18.59	14.36	0.00–53.00	13.50	0.91	0.044	0.01
Self-control	40.66	10.01	16.00–65.00	40.00	41.31	10.69	16.00–65.00	41.00	39.95	9.26	18.00–64.00	40.00	1.21	0.208	0.01
ASR 18/59 Aggressive behavior	6.29	5.60	0.00–25.00	5.00	6.28	5.48	0.00–25.00	5.00	6.36	5.77	0.00–24.00	5.00	2.17	0.884	0.00

M = Mean, SD = Standard deviation. MANOVA was conducted with age as covariate. Subsample sizes were reduced to n = 177 males and n = 170 females in MANOVA

Table 2 illustrates the correlational associations while statistically controlling for age and gender. A higher ACE burden was associated with a higher ADHD severity and elevated aggression ratings, but lower self-control. The ADHD total score showed high positive correlations with aggressive behavior and high negative correlations with self-control. Self-control exhibited moderate negative associations with adult aggression.

**Table 2:** Partial correlations between the variables of interest (bias-corrected and accelerated) with 95% confidence intervals for the total sample

	(1)	(2)	(3)	(4)
MACE total score (1)	–	0.373*** [0.284, 0.450]	− 0.332*** [− 0.429, − 0.223]	0.434*** [0.353, 0.505]
ADHS-SB (2)		–	− 0.751** [− 0.793, − 0.700]	0.720*** [0.662, 0.775]
Self-control (3)			–	− 0.608*** [− 0.661, − 0.542]
ASR 18/59 Aggressive behavior (4)				–

***p < .001, *p* < .05, **. Analyses based on z-standardized values with age and gender as covariates. N = 350

Table 3 displays the correlations between the aforementioned variables, separately for males and females with age as a control variable. The observed correlations were predominantly consistent for both men and women and reflected the results for the total sample. However, significant differences emerged in the gender-specific relationship between ADHD severity and ACEs as well as ADHD severity and adult aggression.

**Table 3:** Partial correlations between the variables of interest (bias-corrected and accelerated) with 95% confidence intervals for males and females

		(1)	(2)	(3)	(4)
MACE total score (1)	Male	–	0.269*** [0.133, 0.403]	− 0.289*** [− 0.415, − 0.165]	0.398*** [0.285, 0.499]
	Female	–	0.442*** [0.301, 0.571]	− 0.365*** [− 0.499, − 0.232]	0.457*** [0.326, 0.571]
	Z	–	− 1.836*	0.786	− 0.667
ADHS-SB (2)	Male		–	− 0.745** [− 0.809, 0.669]	0.668*** [0.551, 0.768]
	Female		–	− 0.760** [− 0.817, − 0.699]	0.757*** [0.671, 0.827]
	Z		–	0.319	− 1.680*
Self-control (3)	Male			–	− 0.583*** [− 0.669, − 0.493]
	Female			–	− 0.631*** [− 0.709, − 0.544]
	Z			–	0.702
ASR 18/59 Aggressive behavior (4)	Male				–
	Female				–
	Z				–

*p < .05, ***p < .001. Analyses based on z-standardized values with age as a covariate. n = 177 for males and n = 170 for females

### Serial mediation analyses

**Fig. 1:**
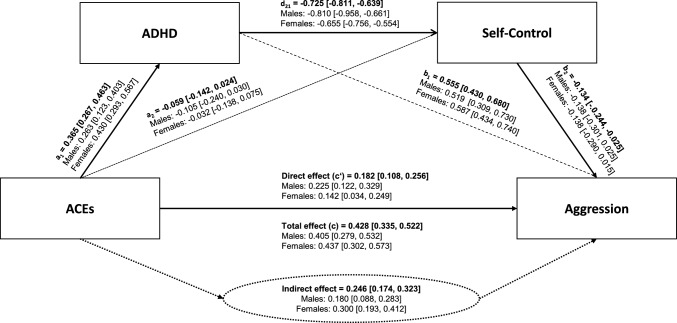
Serial mediation analyses showing the relationship between ACEs and aggressive behavior via ADHD symptom severity and self-control for the total sample (n = 350) and the gender-separated samples (male: n = 177) and (female: n = 170). In the standard model, the analysis was controlled for age and gender. In gender-separated analyses, only age was controlled for. 95% confidence intervals are presented in brackets

Figure 1 presents the serial mediation model illustrating the effect of ACEs on aggressive behavior, with ADHD symptom severity as the first mediator (M1) and self-control as the second mediator (M2) in sequence. The model was analysed for the total sample (with age and gender included as covariates) and, for exploratory purposes, separately for females and males (with age included as a covariate). This standard model accounted for 57.1% of the variance in aggressive behavior (females: 59.9%, males: 54.5%, *p* < 0.001).

ACEs significantly predicted both ADHD severity and aggressive behavior. Furthermore, ADHD severity was significantly associated with both self-control and aggressive behavior. In the serial mediation model, ACEs did not exhibit a significant predictive effect on self-control, whereas self-control significantly predicted aggression. The direct effect of ACEs on aggression was smaller than the total effect. The significant indirect effect indicates that ADHD and self-control sequentially mediate the relationship between ACEs and aggression.

The simple indirect effect of ACEs on aggressive behavior through ADHD (Δ = 0.203, SE = 0.035, 95% CI [0.139, 0.274]) and the indirect effect through the sequence of ADHD and self-control (Δ = 0.036, SE = 0.015, 95% CI [0.007, 0.065]) were both positive and statistically significant, while the indirect effect of ACEs on aggressive behavior via self-control alone (Δ = 0.008, SE = 0.007, 95% CI [− 0.003, 0.025]) was not significant.

When gender-based subsamples were analysed separately, the results largely mirrored those of the total sample, with the exception of non-significant effect in the path between self-control and aggression for both females and males.

## Discussion

Employing a serial mediation model, the current study aimed to investigate the underlying mechanisms by which ACEs influence adult aggression in a mixed adult sample. Our results revealed that the severity of ADHD symptoms and self-control could serve as mediators in the relationship between ACEs and adult aggression, operating in a sequentially mediated manner.

The hypotheses introduced in this study were largely supported in both the overall sample and the gender-separated analyses.

The number of self-reported ACEs was associated with elevated aggressive behavior scores, aligning with prior studies (Burke et al. 2023; Juan et al. 2020; King 2021; Malvaso et al. 2021; Stoppelbein et al. 2024; Woehrle et al. 2022). Notably, the association of ACEs with aggression lost some of its predictive power upon the introduction of ADHD severity and self-control as mediating variables, which highlights the synergistic role of ADHD severity and self-control in the relationship between ACEs and aggressive behavior. In accordance with existing research, the present study demonstrated that ACEs were significantly associated with increased ADHD symptom severity (Craig et al. 2020; Wojtara et al. 2023; Zhang et al. 2022) and that ADHD severity exhibited a robust and positive correlation with aggression (Saylor and Amann 2016). Thus, the dynamics observed in our analysis further underscore the relevance of ACEs in relation to antisocial behaviors in individuals with severe ADHD (Barra et al. 2022).

Furthermore, the present findings align with previous research linking ACEs to lower levels of self-control (Doelman et al. 2021; Stevens et al. 2016; Walters 2018). Regarding the relationship between self-control and aggression (Vazsonyi et al. 2017), our results are consistent with the assumptions of the General Theory of Crime (Gottfredson and Hirschi 1990), though the correlational nature of the data limits causal interpretation.

Previous research has identified moderate impairments in self-control among individuals with ADHD (Patros et al. 2016), a finding that is consistent with the present study. In the Pittsburgh Youth Study, White et al. (1994) examined the relationship between cognitive and behavioral impulsivity, conceptualized as the inverse of self-control, and subsequent delinquent behavior in children exhibiting ADHD symptoms, using self-reports, as well as parent and teacher assessments. Longitudinal analyses provided compelling evidence that both cognitive and behavioral impulsivity significantly predicted antisocial behavior, with statistically robust effects observed in both cross-sectional and longitudinal models.

Stevens et al. (2008) found that symptoms of inattention and hyperactivity were associated with higher levels of disinhibited behavior. Posttraumatic reactions, such as increased arousal and hypervigilance, may contribute to difficulties in stress regulation and anger control. Similarly, Rydelius (1981) and van der Kolk (1997) observed psychomotor agitation, impulsive outbursts, and concentration difficulties in individuals with ACEs. Considering those findings, it is crucial to differentiate whether those symptoms are to be seen in the light of ADHD or rather reflect aspects of post-traumatic stress disorder (PTSD). A recent meta-analysis (Magdi et al. 2025) examining the comorbidity of ADHD and PTSD in adults concluded that a higher prevalence of ACEs and an increased risk of multiple comorbid psychiatric disorders were linked to their co-occurrence. Due to overlapping symptom patterns, the differentiation between ADHD and PTSD in survivors of ACEs can be challenging.

The higher prevalence of ADHD in males compared to females may be attributable to underdiagnosis or delayed diagnosis in females. According to Taylor and Keltner (2002), ADHD in females may be more frequently overlooked due to a higher intelligence quotient, which can obscure or mask symptoms and thus delay recognition and diagnosis. A possible explanatory approach for the higher ADHD scores observed among female participants in our study lies in gender-specific differences in symptom reporting. Women with ADHD more frequently exhibit internalizing symptoms, whereas men tend to display externalizing characteristics (Gaub and Carlson 1997; Gershon and Gershon 2002; Quinn 2008). These internalizing symptoms appear to be particularly reliably captured through self-report measures. Skogli et al. (2013) demonstrate that self-assessment instruments show higher diagnostic sensitivity for internalizing comorbidities in women with ADHD. Given the declining gender ratio in ADHD diagnoses in adults, Hinshaw et al. (2022) also assume that self-reports by women have higher diagnostic accuracy than those by men. This gender-specific measurement sensitivity may have contributed to a systematic overestimation of ADHD symptom burden among female participants in the present study, which primarily relies on self-reported data.

Although previous empirical studies have demonstrated higher rates of ACEs among females (Baglivio et al. 2014; Felitti et al. 1998) the present study did not reveal a significant gender difference. This discrepancy may be explained by the specific composition of the sample. Especially male participants in forensic settings tend to report ACEs more frequently, potentially neutralizing gender differences (Umpunjun et al. 2024). Moreover, the mode of data collection may have influenced the findings. While females may be more open to disclosing certain types of ACEs (Strine et al. 2012), there is evidence of systematic underreporting among male victims of sexual violence and other forms of ACEs, due to a lack of willingness to disclose such experiences (Negriff et al. 2014; Vaswani 2018). Forensic or clinical contexts may facilitate disclosure through structured interviews or psychoeducation, thereby reducing reporting bias. Overall, the present findings suggest that gender differences in the prevalence of ACEs may depend on the context. Their interpretation should always take into account the interaction between sample characteristics, data collection methods, and potential sources of bias.

Contrary to the consistent findings from meta-analyses (Archer 2004; Bettencourt and Miller 1996), which indicate that males, on average, exhibit higher levels of aggression than females, the present study did not reveal a significant gender difference in aggression levels. This result requires careful interpretation, as it may be influenced by specific characteristics of the assessment method employed. In this study, aggression was measured exclusively through self-report instruments. Such methods are susceptible to distortions due to social desirability and gender role norms (Hyde 2014; Serico et al. 2021). In clinical or forensic contexts, for instance, men may tend to downplay aggressive tendencies in order to avoid being perceived as violent or prone to losing control, whereas women may be more open in reporting such experiences. It is also conceivable that gender-specific expressions of aggression, such as direct physical aggression in men and indirect or relational aggression in women, were not adequately captured by the instrument used. When aggression is analysed as a global construct, different forms of aggression may statistically offset one another, resulting in the appearance of no gender difference (Archer 2004; Eagly and Steffen 1986). A more differentiated analysis of aggression subtypes, along with the use of multimethod approaches such as combining self-reports with observer ratings, would be valuable for future research.

In line with previous findings, the present gender-specific results indicate that both women and men exhibit comparable levels of self-control (de Ridder et al. 2012).

The findings of the present study are subject to specific strengths and limitations. Although the sample provides valuable insights into the studied phenomenon, it is based solely on data collected by a single research institute in Germany and, therefore, does not allow for broad generalizability. Therefore, it is necessary to replicate the current findings while considering further critical covariates in a larger sample. Additionally, even if the German 75-item version of the MACE scale captures multiple facets of ACEs, it does not claim to provide a comprehensive assessment of all possible harmful experiences in childhood and adolescence. Moreover, the retrospective nature of data collection entails the risk of multiple biases, as highlighted in previous research (De Sanctis et al. 2012). However, given the sensitive nature of the constructs examined, particularly the retrospective assessment of ACEs, self-reports represent a particularly suitable method for capturing these experiences, although their validity may be limited by recall biases and motivational factors (Breton et al. 2022; Steele et al. 2024). A central methodological strength of the present study lies in the heterogeneous composition of the sample, which allowed for the investigation of associations across a broad spectrum of interindividual differences. However, the inclusion of diverse subgroups (clinical, forensic, and non-clinical/non-forensic populations) without explicitly accounting for group membership could introduce potential biases. Subgroup-specific analyses or statistical adjustments for group membership were not conducted due to insufficient statistical power resulting from the sample sizes within the individual subgroups.

It is also essential to emphasize that the present study did not establish a clinician-administered ADHD diagnosis but focused on self-reported ADHD severity. While the ADHS-SB is a well-validated self-report instrument, it does not provide a sufficient basis for diagnostic clarification on its own but serves as a valuable complement to clinical assessments of ADHD severity (Rösler et al. 2021). However, as Ginsberg et al. (2014) emphasized that ADHD in adulthood is often underdiagnosed and many patients receive insufficient or inadequate treatment, not only focusing on diagnosis but rather symptom severity may be of importance for research and clinical purposes.

Since we did not rely on clinician-administered diagnoses, we could also not include any psychiatric comorbidities. Future research should pay particular attention to the frequently observed and clinically important overlap between ADHD symptoms and trauma-related disorders (Schneider et al. 2019; Szymanski et al. 2011). Moreover, ADHD frequently co-occurs with a range of mental health problems, including affective, anxiety personality, substance use, and obsessive–compulsive disorders (Modesti et al. 2025; Schiweck et al. 2021; Spencer et al. 2007).

Although the serial mediation models applied in the present study were grounded on theoretically well-established assumptions regarding the effects of ACEs, ADHD symptom severity, and the role of self-control in predicting adult aggression, it is crucial to underscore that the data relied on a cross-sectional, correlational design. Consequently, causal interpretations cannot be drawn from the results. To establish ‘true’ mediation, it would be necessary to first provide evidence for the temporal sequence of the effects and establish causality by longitudinal approaches (MacKinnon and Fairchild 2009). The research group led by Craig (2020) highlighted stable cross-sectional findings in the relationship between ACEs and ADHD, whereas longitudinal studies demonstrated less robust associations. Thibodeau et al. (2015) suggested that antisocial behavioral tendencies should not be regarded solely as a consequence of self-control deficits, as these constructs may evolve concurrently. Moreover, less prominent yet significant associations indicated a reciprocal relationship between ACEs and childhood ADHD, but ADHD may also increase the likelihood of experiencing further distressing events later in life (Lugo-Candelas et al. 2021), which we could not include in the present study. These intricate relationships remain an important avenue for additional longitudinal research.

However, future studies should build on the current findings and aim to comprehensively assess ACEs as close in time to the exposure as possible. Investigating the influence of developmental age stages at the time of exposure to ACEs on aggressive behavior in adulthood would be of considerable scientific interest. While existing research suggests the presence of sensitive periods in the etiology of various psychological disorders (Schalinski et al. 2016), comparable evidence regarding aggression remains lacking. Fava et al. (2023) found that the association between ACEs experienced before the age of 11 and delinquency in late adolescence was indirectly mediated by low self-control in middle adolescence.

The present findings underscore the need to implement targeted screening programs for individuals with ADHD, enabling the early identification of at-risk individuals to prevent lifelong maladaptive development (Moffitt 2006). Clinical practice should prioritize early detection of ACEs in this context. Previous scholars claimed that general practitioners often overlooked ACEs, highlighting the need for increased awareness in primary care settings; e.g., a study by Kerker et al. (2016) identified the lack of knowledge among pediatricians regarding the identification of ACEs. According to their findings, 49% of respondents stated that they had only recently become aware of a screening tool for ACEs, and 46% reported that they had never used it. Only 4% used the tool occasionally, and merely 2% incorporated it into their routine practice. The present analyses also allow implications for forensic assessment, as results reinforce the significant role of ADHD for the occurrence of aggressive behavior (Baggio et al. 2018; Freckelton 2019; Retz and Rösler 2009; Young et al. 2015).

Special attention should also be given to protecting and supporting individuals in urgent need of intervention. Self-control training, as well as trauma-focused interventions or disorder-specific treatment approaches, should be promptly implemented while incorporating systemic intervention strategies. Self-control training is an established strategy for behavior modification in which individuals assume primary responsibility for regulating impulsive behaviors while fostering adaptive habits (Zheng 2022). A growing body of research has been dedicated to examining the efficacy of self-control training in alleviating ADHD symptoms (Beh-Pajooh et al. 2012; Salleg-Cabarcas et al. 2024). A pretest–posttest study found that a mindfulness intervention improved self-control and reduced risk behaviors in adolescents with ADHD (Golestaneh et al. 2022).

Despite the extensive body of ADHD and aggression research, our study makes a novel contribution by examining the previously underexplored mediating role of ADHD symptom severity and self-control in the relationship between ACEs and aggression. By integrating theoretical frameworks with empirical evidence, this research enhances the understanding of the complex interplay among these variables, offering a more nuanced perspective on the mechanisms underlying aggressive behavior. These findings might have significant implications for future intervention strategies, emphasizing the need for tailored approaches that aim to reduce the impact of ACEs, foster self-control skills, and implement effective ADHD-focused cognitive behavioral therapy. Pharmacological interventions should be considered alongside psychotherapeutic approaches, with evidence suggesting that such treatment may be effective in reducing recidivism and facilitating rehabilitation among incarcerated individuals with ADHD (Carlander et al. 2024; Lichtenstein et al. 2012). Implementing such targeted interventions may contribute to disrupting the cycle of violence and reducing aggressive behavior in adulthood.

## Supplementary Information

Below is the link to the electronic supplementary material.

Supplementary file1 (DOCX 14 KB)

## Data Availability

The dataset generated and analysed during the current study are available from the corresponding author on reasonable request.
