# Supplementary material for: Mediating roles of attention-deficit/hyperactivity disorder symptom severity and self-control on the relationship between adverse childhood experiences and adult aggression
Source: J Neural Transm (Vienna). 2025 Sep 23;133(3):561–75. doi: 10.1007/s00702-025-03010-1 (PMC12999651; doi:10.1007/s00702-025-03010-1)
Supplement: Supplementary file 1 — Supplementary file1 (DOCX 14 KB) [file 702_2025_3010_MOESM1_ESM.docx]

**Supplement** Reported Types of Adverse Childhood Experiences based on the MACE Subscales

|  | n | % |  |
| --- | --- | --- | --- |
| PPA | 174 | 49.7 |  |
| PVA | 214 | 61.1 |  |
| PNVEA | 206 | 58.9 |  |
| SEXA | 68 | 19.4 |  |
| EN | 268 | 76.6 |  |
| PN | 157 | 44.9 |  |
| WITP | 55 | 15.7 |  |
| WITS | 66 | 18.9 |  |
| PEERE | 216 | 61.7 |  |
| PEERP | 82 | 23.4 |  |
| *Notes*: *PPA (parental physical abuse); PVA (parental verbal abuse); PNVEA* *(parental nonv. emotional abuse); SEXA (sexual abuse); EN (emotional neglect); PN (physical neglect); WITP (witnessed physical violence toward parents); WITS (witnessed violence toward siblings); PEERE (peer emotional violence); PEERP* *(peer physical violence); N = 350.* | | | |
